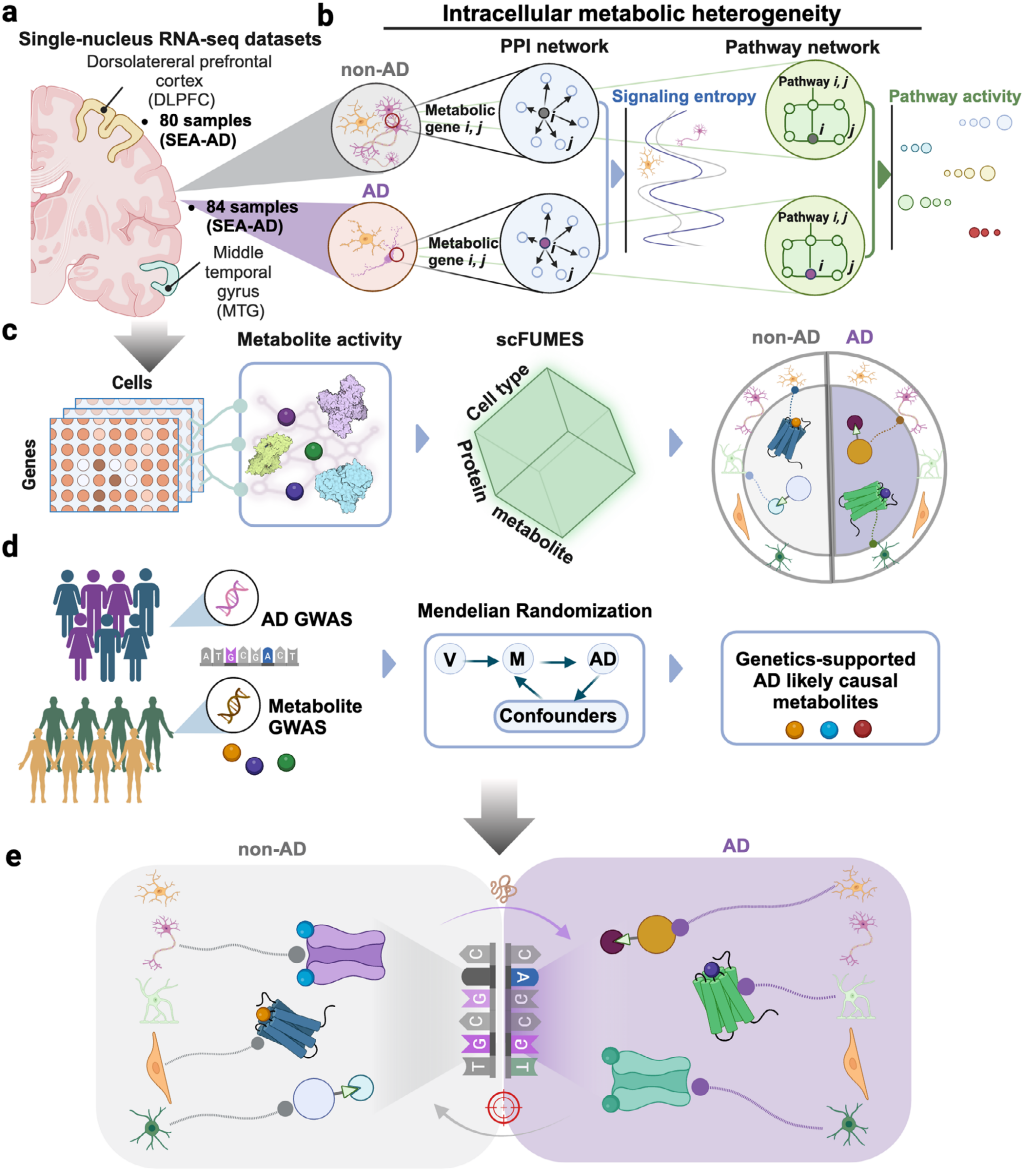# Network‐Based Integrative Single‐Nucleus Multi‐Omics Analysis Reveals Cell Type‐Specific Metabolic Rewiring and Potential Drug Targets for Alzheimer's Disease

**DOI:** 10.1002/alz70855_106203

**Published:** 2025-12-24

**Authors:** Yunguang Qiu, Feixiong Cheng

**Affiliations:** ^1^ Cleveland Clinic, Cleveland, OH, USA; ^2^ Cleveland Clinic Genome Center, Cleveland, OH, USA; ^3^ Case Western Reserve University, Cleveland, OH, USA

## Abstract

**Background:**

Metabolic reprogramming has been implicated as both a cause and consequence of Alzheimer's disease (AD). However, how metabolic signaling dynamics rewire the cellular pathobiology in AD at the single‐cell level is still unclear. This gap in knowledge largely limits our understanding of metabolite‐sensor responses that underpins AD metabolic heterogeneity and metabolism‐based therapeutics development.

**Method:**

We present a multi‐layered omics framework to characterize genetics‐supported metabolite signaling network in specific cellular milieu by integrating large‐scale single‐cell RNA sequencing, genetics, functional/physical measurements, transcriptomics and metabolomics information. This entails four steps: (1) profiling cell‐type‐specific metabolic signaling entropies and pathway activities to evaluate the cellular metabolic heterogeneity in patient's brain with AD. (2) proposing a Single Cell FUnctionally MEtabolite‐Sensor communication (scFUMES) algorithm for predicting metabolic sensing profiles at single‐cell level. (3) Through scFUMES and other omics data, prioritizing AD potential metabolite‐sensor communications to inspect metabolic heterogeneity; (4) evaluating phenotype‐based metabolic signaling and potential AD targets by comparing brain regions, AD severity, sex difference and APOE4 status.

**Result:**

Compared to non‐AD, neuronal cells showed a significant reduction in metabolic signaling entropy and pathway activities in middle temporal gyrus (MTG) and dorsolateral prefrontal cortex (DLPFC) in AD, while most of non‐neural cells have an opposite trend. Particularly, immune cells showed minimal disorder but elevated metabolic activity. Via scFUMES, we prioritized 410 disease‐specific metabolite‐sensor pairs significantly enriched in MTG region. For example, we characterized AD‐risk FABP3‐palmitic acid specific to excitatory neurons and AD‐protective FFAR3‐butyric acid in Oligodendrocytes. Through Mendelian Randomization analysis, we revealed 27 cell‐type specific AD‐associated metabolite‐sensor pairs in MTG. Specifically, we found that a signaling pair KYAT1‐Indole‐3‐propionic acid (a human gut metabolite), is significantly enriched in excitatory neurons in severe AD. We further identified multiple signaling pairs specific in immune cells, such as VDR‐arachidonic acid and ESR1‐L‐phenylalanine. Moreover, sex differences and APOE4 genotypes also exhibited distinct metabolic dynamics in different brain regions, such as PPARD‐glycerol, which is specific in female and non‐APOE4 individuals in immune cells.

**Conclusion:**

The findings systematically reveal a circulating metabolite‐mediated signaling rewiring network, which may shed light on cellular metabolic heterogeneity and cellular metabolism‐based therapeutics for AD and other AD‐related dementia if broadly applied.